# Sialic acids as cellular markers of immunomodulatory action of dexamethasone on glioma cells of different immunogenicity

**DOI:** 10.1007/s11010-018-3478-6

**Published:** 2018-11-15

**Authors:** Przemyslaw Wielgat, Emil Trofimiuk, Robert Czarnomysy, Jan J. Braszko, Halina Car

**Affiliations:** 1 Department of Clinical Pharmacology, Medical University of Bialystok, Waszyngtona 15A, 15-274 Bialystok, Poland; 2 Department of Synthesis and Technology of Drugs, Medical University of Bialystok, Kilińskiego 1, 15-089 Bialystok, Poland

**Keywords:** Glioma, GL261, SMA560, Dexamethasone, Sialic acid, Siglec, Immunogenicity

## Abstract

Glucocorticosteroids, including dexamethasone (Dex), are commonly used to control tumor-induced edema in the brain tumor patients. There are increasing evidences that immunosuppressive action of Dex interferes with immune surveillance resulting in lower patients overall survival; however, the mechanisms underlying these actions remain unclear. Changes in the expression of sialic acids are critical features of many cancers that reduce their immunogenicity and increase viability. Sialoglycans can be recognized by CD33-related Siglecs that negatively regulate the immune response and thereby impair immune surveillance. In this study, we analysed the effect of Dex on cell surface sialylation pattern and recognition of these structures by Siglec-F receptor in poorly immunogenic GL261 and immunogenic SMA560 glioma cells. Relative amount of α2.3-, α2.6- and α2.8-linked sialic acids were detected by Western blot with MAA (*Maackia amurensis)* and SNA (*Sambucus nigra*) lectins, and flow cytometry using monoclonal antibody anti-PSA-NCAM. In response to Dex, α2.8 sialylation in both, GL261 and SMA560 was increased, whereas the level of α2.3-linked sialic acids remained unchanged. Moreover, we found the opposite effects of Dex on α2.6 sialylation in poorly immunogenic and immunogenic glioma cells. Furthermore, changes in sialylation pattern were accompanied by dose-dependent effects of Dex on Siglec-F binding to glioma cell membranes as well as decreased α-neuraminidase activity. These results suggest that glucocorticosteroid-induced alterations in cell surface sialylation and Siglecs recognition may dampen anti-tumor immunity, and participate in glioma-promoting process by immune cells. Our study gives new view on corticosteroid therapy in glioma patients.

## Introduction

Gliomas are the most common primary brain tumors characterized by high pharmacological resistance and poor prognosis [1, 2]. The aggressive potential of glial tumors is attributed to altered expression of genes that control cell signaling, cytoskeletal and receptor proteins as well as cell migration [3–6]. Analysis of gene profiles confirmed also down regulation of MHC I/II proteins expression and increased production of glioma-derived immunosuppressive factors which reverse immune response mechanisms [7, 8]. There are increasing evidences that weak immune surveillance of various cancers, including gliomas, correlates with altered sialylation in malignant cells [9–12]. Sialic acids are nine carbon monosaccharides that occupy terminal positions on glycans through α2.3-, α2.6- and α2.8-linkage, regulate glycoconjugates structure and stability as well as participate in cell–cell and cell–extracellular matrix interactions, including immune recognition [13]. The aberrantly expressed sialic acids reduce cancer immunogenicity by masking of cell surface antigens, recruiting of plasma factor H to control of alternative complement pathways and protecting from clearance by liver receptors [14]. The tumor immune evasion is also facilitated by immune receptor families, such as Siglecs, that recognize cancer sialoglycans and transmit immunosuppressive signals resulting in negative regulation of immune response [13, 15]. The altered sialylation status in malignant cells results from genetic mutations in enzymes that control processing and degradation of bound sialic acids in glycoproteins and glycolipids [16–18]. Additionally, numerous chemical and physical factors are known as modulators of sialotransferases and sialidases activities resulting in hyposialylation or hypersialylation of cellular membranes [19–21].

Dexamethasone (Dex) is a potent steroid commonly used to control tumor-linked edema and radio- and chemotherapy-induced side effects in brain cancer patients [22]. Although Dex is considered as the “gold standard” in glioma therapy for decades, there are growing evidences that effects of corticosteroids on glioma cell growth and patient survival are controversial [23]. The data from mouse glioma models and retrospective clinical analysis showed that Dex may decrease tumor cell proliferation without affecting glioma cell viability and induce gene expression correlated with shorter survival [24]. Both preclinical and clinical observation revealed that Dex may interfere with function of local immune cells resulting in potentiation of glioma-induced weak immunosurveillance. The immunosuppressive actions of corticosteroids can be exerted by nongenomic mechanisms related to interaction with intracellular proteins and modulation of cell membrane adhesion proteins and antigens implicated in the immune recognition [25]. Based on these observations, we evaluated the effect of Dex on sialylated *N*-glycans and *O*-glycans profiles and their recognition by Siglec-F immune receptor. Using glioma cell lines of different histological origin and immunogenicity, we present evidences that Dex-induced changes in cell surface sialylation may interfere with immunogenic potential of glioma cells. Our results emphasize the role of sialic acids in tumor biology and identify Siglec-F as potential important player in glioma immune surveillance during Dex therapy.

## Materials and methods

### Cell cultures and treatment

Both, GL261 (ACC802, DSMZ Germany) and SMA560 (provided by Prof. Neumann, University of Bonn) cells were plated in 6 well plates at a seedinig density 2 × 10^5^/9 cm^2^, cultured in DMEM/F12 medium containing 10% fetal bovine serum and 1% antibiotics, and incubated in 37 °C in a humified atmosphere containing 5% CO_2_. When cells reached confluence at 70–80%, Dex (Dexaven, Jelfa Poland; 0.1 µM, 1 µM, 10 µM) was applied to cell cultures for 24 h. Concentrations of Dex was selected based on previous studies [26].

### Cell cycle

After 24 h of Dex treatment, cells were carefully harvested by scraping, fixed in ice-cold 70% methanol, treated with ribonuclease and stained with propidium iodide (PI, 50 µg/ml) for DNA quantification. The distribution of PI fluorescence in cells was quantified using flow cytometry by their distribution in G0/G1, S and G2/M phases. The S + G2/M population was quantified as proliferating cells.

### Determination of Olig2 expression in GL261 and SMA560 cells

The Olig2 protein is transcription factor known to be required for proliferation of glial tumors and regulated by several sialoglycan expression [27, 28]. Naïve and Dex-treated cells were scraped, diluted to 10^5^ per sample and incubated with Olig2 (Abcam, 2,5 µg/ml) antibody for 30 min at 4 °C. To facilitate intracellular staining, 0.01% Triton was used. Cells were washed with phosphate buffered saline, stained with appropriate secondary fluorescent antibody and analysed on Becton Dickinson flow cytometry system. In each analysis, corresponding isotype control antibody was used as a negative control.

### Assessment of α2,3- and α2,6- and α2,8-sialylation in glioma cells

The α2,3- and α2,6-linked sialic acids in GL261 and SMA560 cells were analysed using the DIG Glycan Differentiation Kit (Roche, Germany) following the manufacturer’s instructions. Terminal sugar structures were recognized by *Maackia amurensis* agglutinin (MAA) and *Sambucus nigra* agglutinin (SNA), that bind α2,3- and α2,6-linked sialic acids, respectively. For Western blot, naïve and Dex-treated cells were homogenized in RIPA buffer containing proteases inhibitors. Twenty micrograms (20 µg) of protein from cellular homogenates were loaded into 10% SDS-polyacrylamide gel, electrophoresed and transferred to PVDF membrane. Blots were incubated with digoxygenin-labeled lectins at 4 °C overnight and anti-digoxygenin Fab fragments conjugated with alkaline phosphatase for 1 h at room temperature. Immunoreactive sialoglycoproteins were visualized with BCIP/NBT Liquid Substrate System (Sigma Aldrich) for alkaline phosphatase. Membranes were scanned and analysed densitometrically using Quantity One (Bio-rad Laboratories, Inc.) and ImageJ softwere. Protein concentration in each sample was estimated by the method of Bradford using bovine serum albumin as a standard [29]. To estimate the level of α2.8-sialylation, cells were analysed by flow cytometry after incubation with primary PSA-NCAM antibody (Merck, 2 µg/ml) for 30 min at 4 °C and staining with appropriate secondary, isotype specific FITC-conjugated antibody (Abcam, 2 µg/ml). In each analysis, corresponding isotype control antibody was used. The amount of PSA-NCAM was determined according to isotype control antibodies used as negative control (Abcam, 2 µg/ml).

### Determination of Siglec-F binding to glioma cells

To assess the binding of Siglec-F protein to glioma cells, the control and Dex-treated cells were incubated with recombinant mouse Siglec-F/Fc Chimera (R&D Systems, 1 µg/ml) and then stained with Cy3 conjugated IgG secondary antibody (Jackson ImmunoResearch, 2 µg/ml). Samples were analysed by flow cytometry and cells stained using the secondary antibody alone were used as negative control. Sialic acid-dependent binding of Siglec-F was confirmed using α-neuraminidase. Briefly, the growing cells were incubated with α-neuraminidase (100 U/ml, from *Clostridium perfringens*, New England Biolabs) for 24 h at 37 °C.

### α-Neuraminidase activity assay

The total Neu activity was assayed using Amplex Red Neuraminidase Assay Kit (Invitrogen) following manufacturer’s instruction. In brief, 50 µl of diluted cell homogenate containing equal protein amount (30 µg) was incubated with 50 µl of Amplex Red working solution comprising 100 µM Amplex Red reagent; 0.2 U/ml horseradish peroxidase; 4 U/ml galactose oxidase and 500 µg/ml fetuin. The activity was measured as absorbance and read at 560 nm using BioTek EL800 microtiter plate reader.

### Statistical analysis

For each group, a minimum of 3–5 independent experiments were studied. The statistical analysis was performed using one way ANOVA followed by Bonferroni post-test. Results are expressed as mean ± SD. Significant differences were deemed at *p* < 0.05.

## Results

### Dexamethasone treatment effects on glioma GL261 and SMA560 cells growth

**Fig. 1:**
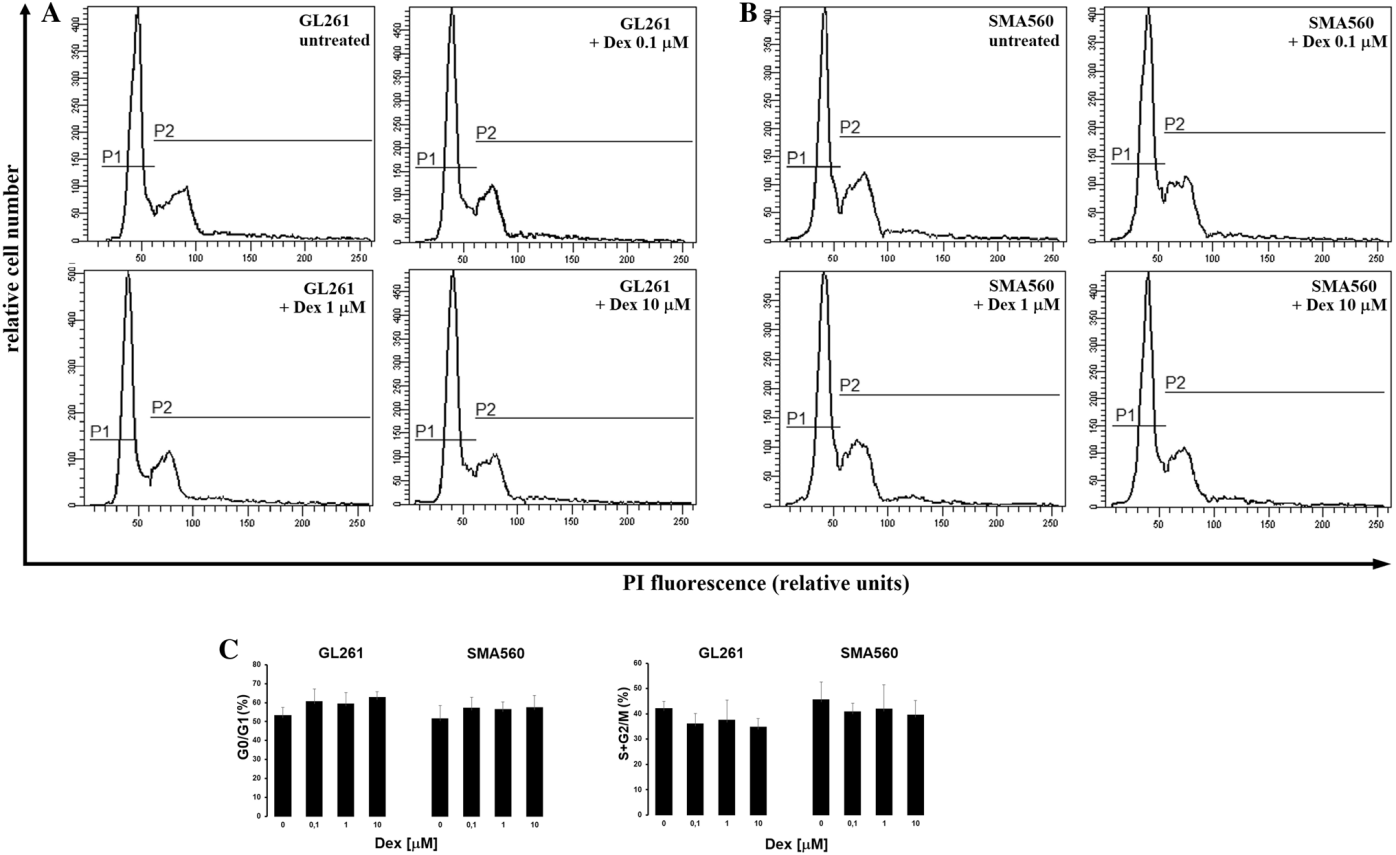
Dose-dependent effects of Dex on the cell cycle phase distribution of GL261 (**a**) and SMA560 (**b**) cells. Representative histograms and corresponding bar graphs (**c**) were derived from 10,000 cells and present the percentage of population within the G_0_/G_1_ (P1) and S + G_2_/M (P2) phases of cell cycle. Each data point is a mean of 3 independent experiments

The exposure to Dex for 24 h induced changes in the distribution of glioma cells along the G_o_/G_1_ and S + G_2_/M cycle phases, but these effects were relatively weak. In GL261 cells, the increasing concentrations of Dex caused enhancement in the number of cells within the G_o_/G_1_ phase, from 52% of the naïve cell population to 61.6% of cells treated with 10 µM Dex. The percentage of proliferating cells within S + G_2_/M phase was correspondingly decreased from 41.3% of the untreated GL261 cells to 33.9% cells exposed to the higher concentration of Dex (Fig. 1a, c). In SMA560 cells, exposure to Dex for 24 h caused enhancement in the number of cells displaying G_0_/G_1_ phase and correspond decrease of percentage of the population within S + G_2_/M phase, but these effects were weak when compared to untreated cells (by 6%; Fig. 1b, c).

### Effects of dexamethasone on Olig2 expression in GL261 and SMA560 cells

**Fig. 2:**
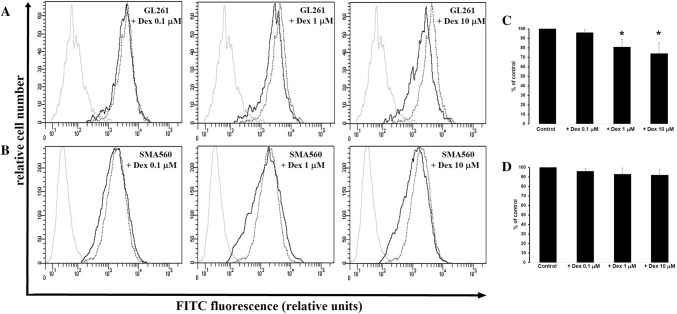
Expression of Olig2 in GL261 (**a**) and SMA560 (**b**) cells exposed to Dex. Representative histograms were derived from analysis of 10,000 cells and show isotype control (light grey line); control cells (dropped line) and cells treated with Dex (black line). **c, d** each column presents mean ± SD of 3–5 independent experiments. Data are presented as a percentage of control group (100%); *p < 0.05 versus control

In additional quantification of anti-proliferatory Dex effects, we analysed the expression of Olig2 known as a transcriptional regulator of proliferation and glioma tumorigenesis. Both, control proliferating GL261 and SMA560 cells expressed high level of Olig2. In GL261 cells, the Olig2 expression was significantly decreased at Dex concentration of 1 µM and 10 µM, but not 0.1 µM, after 24 h of treatment compared to control (0.1 µM Dex: 95.4 ± 3.3% vs. 100% control; 1 µM Dex: 80.6 ± 8% vs. 100% control; 10 µM Dex: 74 ± 11,8% vs. 100% control). In SMA560 cells, the treatment with increasing concentrations of Dex, resulted in slight decrease of Olig2 expression (0.1 µM Dex: 96 ± 3.2% vs. 100% control; 1 µM Dex: 91.8 ± 6.5% vs. 100% control; 10 µM Dex: 92 ± 5.8% vs. 100% control; Fig. 2).

### Effects of dexamethasone treatment on sialylation of glioma cells

**Fig. 3:**
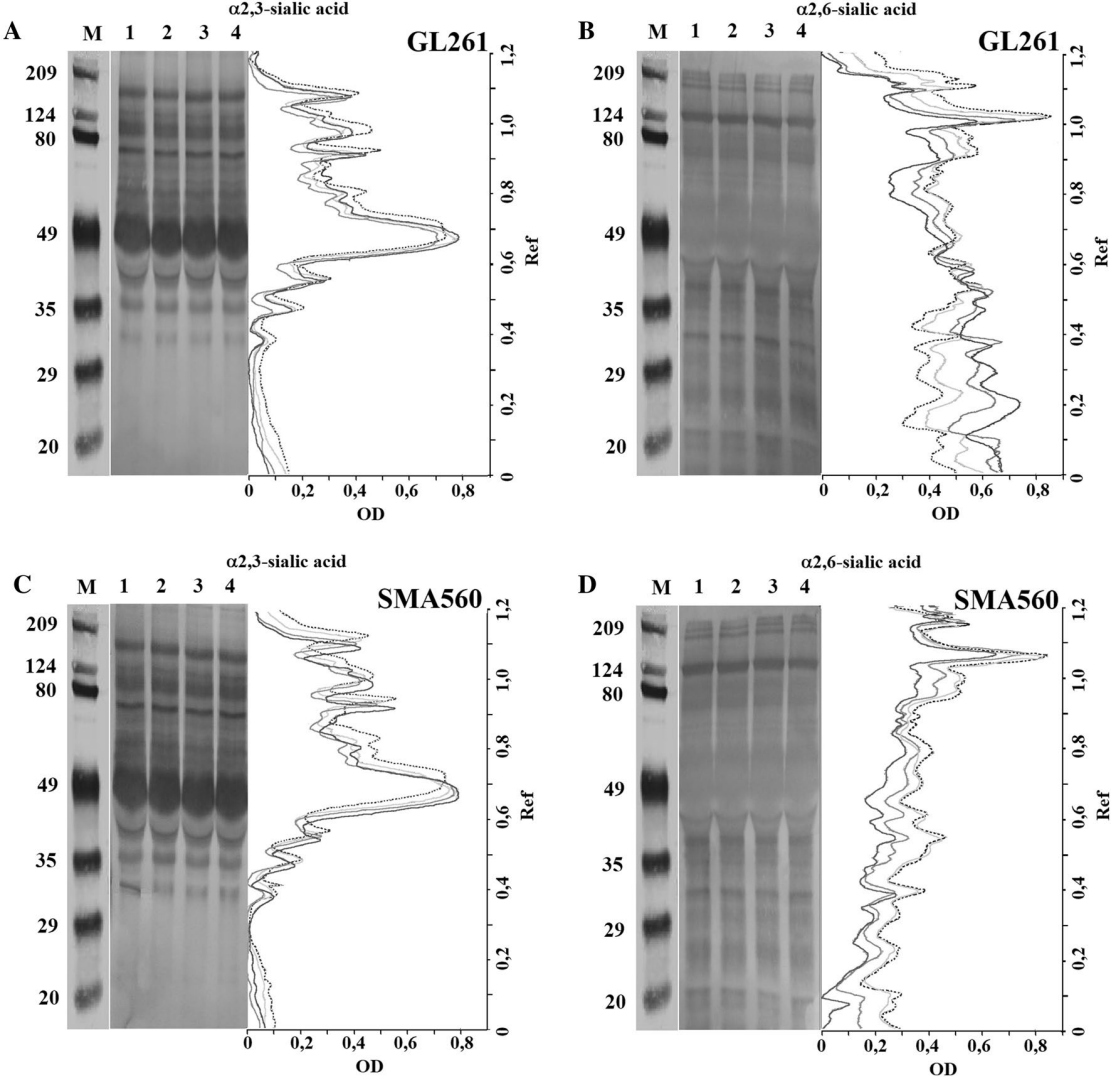
Sialoglycans in GL261 and SMA560 cells exposed to Dex. Representative Western blots of **a** α2,3- and **b** α2,6-sialylated glycoconjugates and corresponding density histograms are shown (dropped line—control cells; light grey line—Dex 0.1 µM; grey line—Dex 1 µM; black line—Dex 10 µM). Lanes show: *M* molecular weight standards, *1* control cells, *2* Dex 0.1 µM, *3* Dex 1 µM, *4* Dex 10 µM

**Fig. 4:**
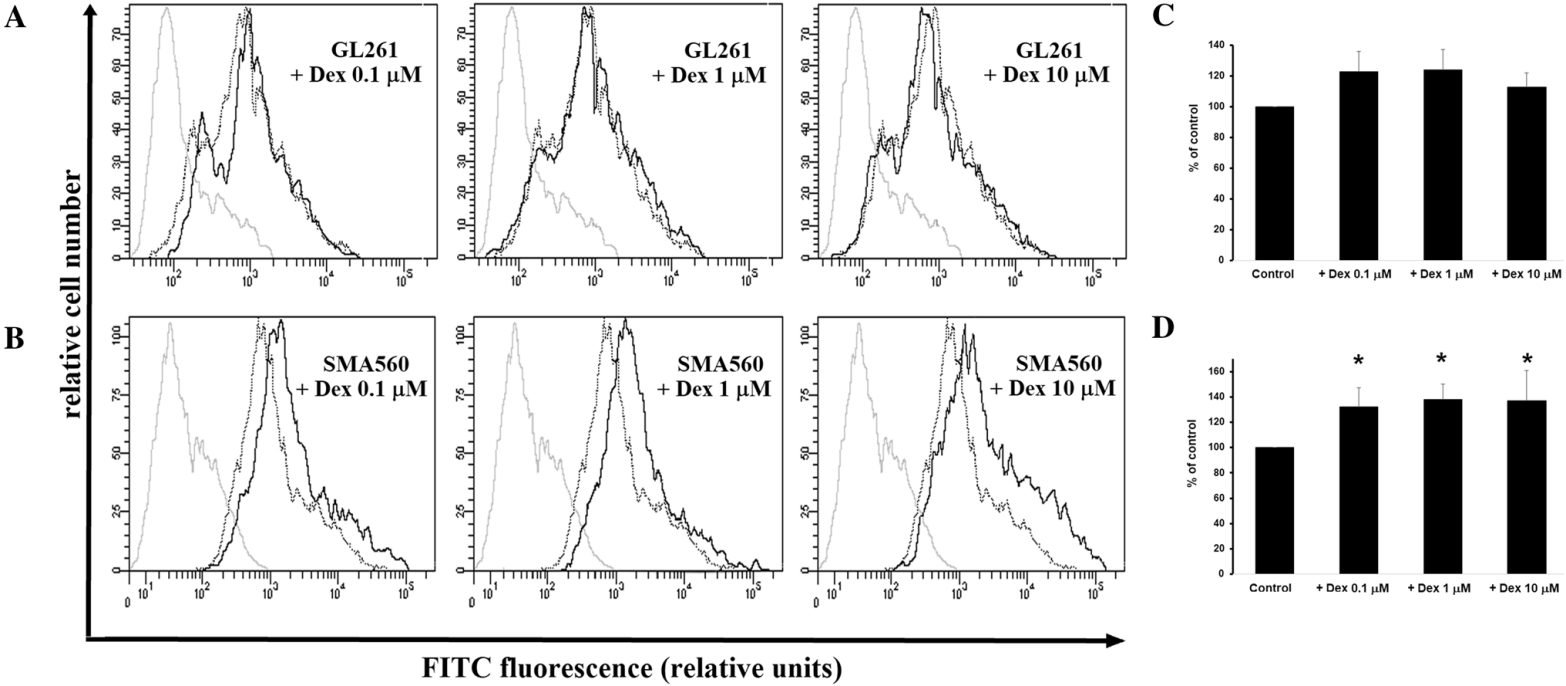
Flow cytometric analysis of PSA-NCAM containing α2,8-linked sialic acids in GL261 and SMA560 cells after exposure to Dex. Representative histograms (**a, b**) were derived from analysis of 10,000 cells and show isotype control (light grey line); control cells (dropped line) and cells exposed to Dex (black line). **c, d** each column presents mean ± SD of 3–5 independent experiments. Data are presented as a percentage of control group (100%); *p < 0.05 versus control

To visualize α2.3- and α2.6-sialylation pattern, the proteins from cellular homogenates were separated on SDS-PAGE, electroblotted and labelled with appropriate lectins. Representative blots and corresponding density bars presented in the Fig. 3 illustrate the amount of terminal linkage-specific sialic acids on cell surface glycoconjugates. In response to increasing concentrations of Dex, α2.3-sialylation determined by the level of reactivity for MAA lectin was remained unchanged in both GL261 and SMA560 cells compared to naïve cells (Fig. 3A, C). The not significantly higher reactivity of MAA lectin with α2.3-linked sialic acids in GL261 and SMA560 cells was observed in molecular weight around 49 kDa at all tested concentrations of Dex (Fig. 3a, c). The α2.6-sialylation in SMA560 cells was decreased across the full molecular weight range as compared to naïve population (Fig. 3d). In contrast with SMA560 cells, the reactivity of SNA lectin in GL261 cells was differentially regulated by increasing concentrations of Dex. The densitometric measurement of selected lanes showed reduced α2.6-sialylation of glycoconjugates in the molecular weight above 49 kDa, but strongly enhanced under 49 kDa (Fig. 3b). The level of α2.8-sialylation was assessed by flow cytometric measurement of polysialic acid posttranslationally attached to NCAM (PSA-NCAM). The effects of increasing concentrations of Dex on PSA-NCAM expression in SMA560 cells were opposite to those seen in α2.6-sialilation of these cells. After 24 h of treatment with Dex, PSA-NCAM expression in SMA560 cells were significantly enhanced to: 0.1 µM: 132.2 ± 15.2% vs. control (100%); 1 µM: 138 ± 12.1% versus control (100%); 10 µM: 136.3 ± 23.6% versus control (100%; Fig. 4b, d). The treatment of GL261 cells with increasing concentrations of Dex for 24 h caused statistically insignificant increase in PSA-NCAM expression as follows: 0.1 µM: 122.7 ± 7.9% versus control (100%); 1 µM: 124 ± 3.2% versus control (100%); 10 µM: 113.3 ± 9.2% vs. control (100%; Fig. 4a, c).

### The binding capacity of Siglec-F/Fc Chimera to glioma cells

**Fig. 5:**
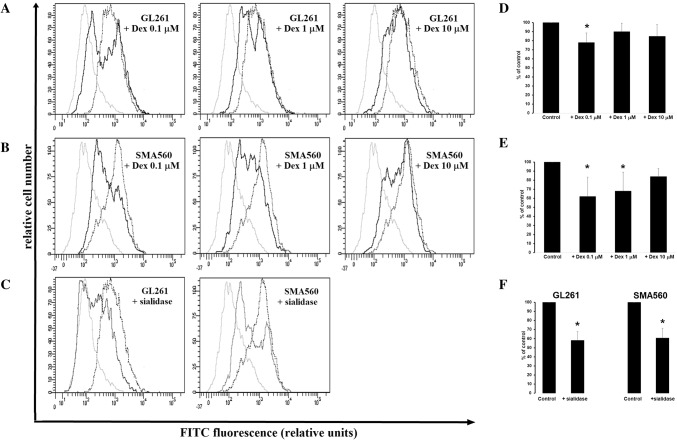
The binding of Siglec-F/Fc Chimera to GL261 (**a**) and SMA560 (**b**) glioma cells. Representative histograms were obtained from flow cytometric analysis of 10,000 cells and show isotype control (light grey line); control cells (dropped line) and cells exposed to Dex (black line). **d, e** each column presents mean ± SD of 3–5 independent experiments. The histograms (**c**) and appropriate bar graphs (**f**) showing cells treated with α-neuraminidase used here as positive control are also included (light grey line); control cells (dropped line) and α-neuraminidase treated cells (black line). Data are presented as a percentage of control group (100%); *p < 0.05 versus control

The analysis of Siglec-F/Fc Chimera positive cells, expressed as a mean relative fluorescence intensity, evidenced differences between control and Dex-treated groups. The measurement of the effect of α-neuraminidase, used here as a positive control, showed signifficant reduction of Siglec-F/Fc Chimera binding to GL261 and SMA560 cells by 42 ± 9.7% vs. control (100%) and 40 ± 10.9% vs. control (100%), respectively (Fig. 5c, f). Dex at all used concentrations reduced the binding capacity of Siglec-F/Fc protein to both GL261 and SMA560 cells. In details, the mean fluorescence intensity of SMA560 cells was significantly decreased at Dex concentration of 0.1 µM and 1 µM but 10 µM, after 24 h of treatment compared to control (0.1 µM Dex: 62 ± 21.5% vs. 100% control; 1 µM Dex: 68 ± 20.8% vs. 100% control; 10 µM Dex: 84 ± 8.8% vs. 100% control; Fig. 5b, e). When GL261 cells were exposed to Dex, the affinity of Siglec-F/Fc protein tended to be reduced, but differences were not significant at concentration of 1 and 10 µM (0.1 µM Dex: 78 ± 10.7% vs. 100% control, *p* < 0.05; 1 µM Dex: 90 ± 9.5% vs. 100% control; 10 µM Dex: 85 ± 12.7% vs. 100% control); Fig. 5a, d.

### Effects of dexamethasone on α-neuraminidase activity

**Fig. 6:**
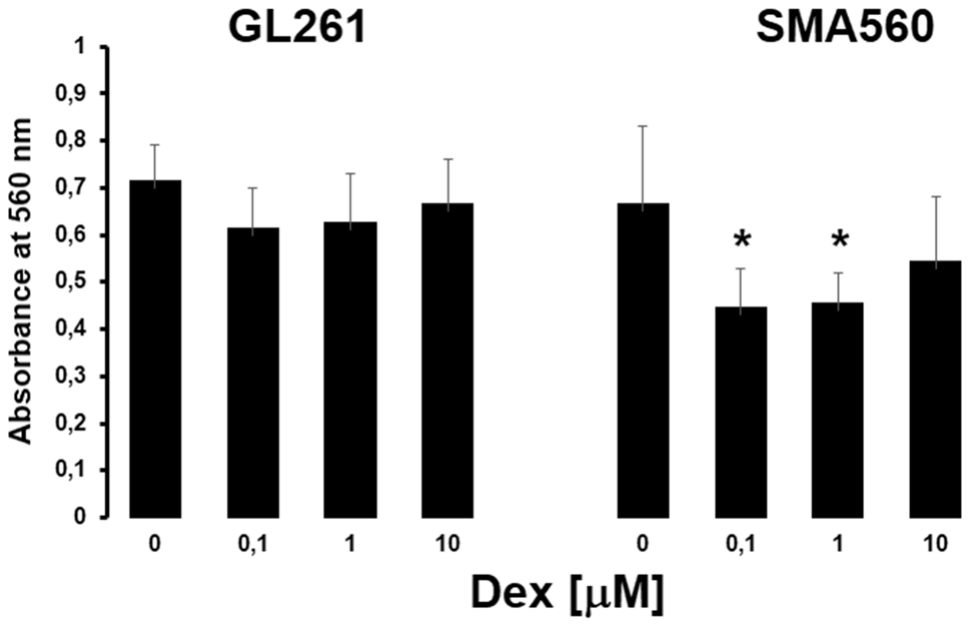
Detection of neuraminidase activity using the Amplex Red Neuraminidase Assay Kit in GL261 and SMA560 cells treated with Dex. Absorbance at 560 nm is shown. Each column presents mean ± SD of 3 independent experiments; *p < 0.05 versus control

To confirm that Dex exerts dose-dependent changes in glioma cell sialylation, we assessed the activity of α-neuraminidase which is closely associated with sialoglycans turnover. Our experiment with the treatment of GL261 and SMA560 cells with increasing concentrations of Dex for 24 h showed that α-neuraminidase enzymatic activity was decreased in both cell lines (Fig. 6). In GL261 cells, enzymatic activity decreased by 15%, 13% and 8% at Dex concentration of 0.1 µM; 1 µM and 10 µM, correspondingly, compared to control group. The neuraminidase activity decreased significantly in SMA560 cells by 31% (*p* < 0.05), 33% (*p* < 0.05) and not significantly by 19% at Dex concentration of 0.1 µM; 1 µM and 10 µM, respectively .

## Discussion

As mentioned in the introduction, several studies closely connect the aberrant sialylation to tumor immune evasion. Corticosteroids, since they are known as potent modulators of cell biology, stimulate malignant cells and cancer-related immune processes have been investigated in several clinical studies and experimental models. In this work, we hypothesized that Dex involvement in immune surveillance is regulated by mechanisms linked to changed sialic acids and their recognition by Siglecs. We used glioma cells of different immunogenicity, as determined in independent studies based on expression of MHC proteins and immune activity in vivo and in vitro [7]. The GL261 cells are poorly immunogenic due to low expression of MHC Class I/II molecules. In contrast, the SMA560 cells present MHC Class I, which enhances their recognition by effector immune cells [30–32]. Comparison of sialylation pattern in analysed naïve glioma cells indicated that the level of α2.3- and α2.8-sialylation was similar in both naïve GL261 and SMA560 cells, whereas α2.6-sialylation was higher in GL261 cells. This is perhaps because the glioma cell lines may be significantly different in their expression and activity of regulatory mechanisms of cell surface sialylation, and adhesion molecules that promote their malignant phenotypes. Secondly, elevated degree of sialylation in GL261 cells may reflect reduced immunogenicity as described previously in various pathologies [14]. The aberrant α2.6-sialylation correlates with tumorigenesis and tumor progression, and the elevated presence of α2.3-sialic acid residues constitutes a typical feature of tumor adhesion and invasion [33–35]. The exposure of glioma cells to Dex exerted dose-dependent changes in α2.3-; α2.6- and α2.8-sialoglycotopes. Our results indicated that the degree of ɑ2.8-sialylation was elevated in poorly immunogenic cells, and strongly increased in immunogenic glioma cell line. In the same experimental conditions, the expression of α2.3-Sia glycotopes among selected stimulated cells remained unchanged or increased compared to naïve cells, but these effects were relatively weak. Interestingly, the expression of α2.6-Sia residues was higher in poorly immunogenic cells and reduced in immunogenic cell line. In malignant cells, the sialylation is altered by sialyltransferases and sialidases dysregulation, which is distinctive feature of each cancer. It is conceivable, that elevated level of α2.6-Sia glycotopes in GL261 cells results from aberrant expression and activity of α2.6-sialyltransefase (α2.6-ST), which regulates malignant phenotype of these cells. Several studies have demonstrated that expression and activity of α2.6-ST was induced after concentration dependent corticosteroid stimulation in a number of different tissues, including cancerous [36, 37]. In this study, subsequent examination of individual sialylation regulatory enzymes demonstrated strong decrease of total sialidase activity following dexamethasone exposure in SMA560 cells but not in GL261. Since we showed participation of lysosomal glycosidases in human gliomas progression and modulatory effects of corticosterone on sialidases activity in rodent brain, it is reasonable to suggest their crucial role in glioma biology, including immunogenicity. The Dex-induced changes in cell membrane sialylation can be related to differences in glycoproteins structure during cell cycle. Our results suggest that Dex inhibited proliferation of both GL261 and SMA560 by G_0_/G_1_ phase arrest in cell cycle. Moreover, the most effective Dex concentration decreasing Olig2 expression—10 µM—corresponded to that revealed as the most arresting G_0_/G_1_ phase in GL261 and SMA560 cells. It has been shown previously that the total content of several monosaccharides is at minimum, whereas the degree of sialylation is at maximum just before and during cell division glycotopes [38]. It is in agreement with our observation that reduced sialylation correspond to Dex-induced growth inhibition in immunogenic SMA560 cells. In contrast, partially immunogenic GL261 cells showed cell cycle arrest in G0/G1 phase after exposure to Dex and increased level of α2.6-Sia. Similar changes were described by Glick et al. in slow growing cells extendend in G1 phase [39]. Since selected cell lines were tested in the same experimental conditions, the differences in sialylation degree were probably due to their phenotype, which can be evaluated by expression and distribution glioma of proliferation and tumorigenesis markers.

The ability of cancer to induce the host anti-tumor immune response depends on defence mechanisms in resident and infiltrating immune cells that are activated by tumoral soluble factors or during cellular interactions [40–42]. Sialic acids in tumor cells form ligands for CD33-related Siglecs which trigger suppressive signalling to immune cells via tyrosine-based inhibitory motifs (ITIM) and SHP1/SHP2 molecules that modulate cytotoxic and inflammatory responses leading to increased pathology development [43, 44]. Siglec-F, inhibitory CD33-related sialic acid receptor, is highly expressed on murine eosinophils, macrophages and CD4-positive T cells, which are a part of complex glioma microenvironment [45, 46].

As we showed in this study, the naïve glioma cells express high level of sialic acids. They cover tumoral antigens and exert masking effects that facilitate escape from recognition by immune cells [11]. In multivariate analyses, enhanced level of polysialylated NCAMs (PSA-NCAMs) was an independent negative predictor of overall survival of patients with GBM [27]. Besides, sialylated glycans form ligands for CD33-related Siglecs which activate the cellular signalling pathways via tyrosine-based inhibitory motifs (ITIM) and SHP1/SHP2 molecules resulting in cellular inhibition [47]. In this study, sialoglycans of GL261 and SMA560 cells showed high reactivity with recombinant Siglec-F protein. Furthermore, the binding of recombinant Siglec-F protein to glioma cells was strongly reduced by the lowest concentration of Dex, whereas the highest dose had a minimal effect on this process. The effects produced by various doses of Dex were more intense in immunogenic compared to poorly immunogenic glioma cells. This finding may reflect high expression of 6′-sulfated sialyl Lewis X (6′-su-sLeX), which was described as endogenous and inducible ligand for Siglec-F [48–50]. The expression of 6′-su-sLeX and sulfotransferase keratin sulfate galactose 6-*O*-sulfotransferase (KSGal6ST), an enzyme required for its synthesis, were detected in various cancers, including gliomas, and is routinely used as a marker for diagnosis, grading and prognosis [51, 52]. The crosstalk between glioma and immune cells via sialic acid—Siglec-F connection support tumor-promoting functions, including angiogenesis, proliferation, remodeling of extracellular matrix and recruitment of immunosuppressive myeloid cells [53]. Engblom et al. showed that presence of Siglec-F—positive neutrophilia within tumor promotes cancer growth and correlates with poor prognosis [54]. In contrary, eosinophilia in GBM patients correlates with longer survival but Siglec-F—dependent apoptosis of eosinophils appears to be negative prognostic factor [45]. Furthermore, human Siglec-7 on NK cells shows strong binding prevalence for α2.8-linked sialic acids, and thereby leads to an inhibition of these cells cytotoxicity. In this way, Siglec-7 can potentially dampen anti-tumor immunity and promote cancer invasion [55]. Additionally, it has been demonstrated previously, that glucocorticosteroids increase expression of several Siglecs in immune cells in vivo [56, 57]. Given the importance of sialylation and Siglecs in immunity it is reasonable to speculate that Dex can be crucial factor regulating immunogenic potential of gliomas and immunosuppressive phenotype and survival of tumor-associated immune cells. It is in line to several clinical trial observations that higher doses of steroids were negative prognostic factor in patients with large glial tumors and more prominent neurological deficits [24, 58]. The explanation of modulatory role of Dex in sialic acid—dependent immunogenicity requires the analysis of human cell populations implicated in glioma progression. The comparison of sialylation pattern and Siglec-related changes in gliomas and tumor—associated cells could help to evaluate the immune surveillance during Dex therapy and develop new strategies based on changes of Siglecs function and their sialylated ligands.

In conclusion, this study showed that Dex alter both GL261 and SMA560 sialoglycans, but the corresponding differences between their sialylation pattern suggest that these steroid-induced changes in glioma cells of different immunogenicity are not identical. The relationship between changes in sialic acids and their recognition by Siglecs as well as widely known effects of steroids, in particulary high doses of Dex, in glioma prognosis and patients survival suggests the existence of sialic acid-based mechanisms that regulate functional alterations in cancer immunosurveillance.
